# Head Posture and Postural Balance in Community-Dwelling Older Adults Who Use Dentures

**DOI:** 10.3390/medicina56100529

**Published:** 2020-10-12

**Authors:** Youngsook Bae, Yongnam Park

**Affiliations:** 1 Department of Physical Therapy, College of Health Science, Gachon University, Incheon 21936, Korea; baeys@gachon.ac.kr; 2 Department of Physical Therapy, Suwon Women’s University, 1098, Juseok-ro, Bongdam-eup, Hwaseong-si, Gyeonggi-do 18333, Korea

**Keywords:** balance, denture use, forward head posture, older adult

## Abstract

*Background and objectives*: Tooth loss and consequent denture use and impaired posture and postural balance are more prevalent in older adults than in the young ones. The aim of this cross-sectional study was to identify the association between denture use, head posture, postural balance, and neck muscle strength (NMS). *Materials and methods*: We included 107 participants (56 in the non-denture use group and 51 in the denture use group) and measured their NMS, forward head posture, and postural balance. Forward head posture was measured using the craniocervical angle (CRA). Postural balance was assessed using a timed up-and-go test (TUG) and postural sway. An independent t-test was used to analyze the differences between the groups; Pearson correlation analysis was used to analyze the correlation of period of denture use, head posture, and postural balance. *Results*: We found that the denture use group had lower NMS, smaller CRA, longer TUG, and longer postural sway length than the non-denture use group. Duration of denture use was significantly correlated with TUG. *Conclusions*: Our findings reveal that denture use does not help with NMS, forward head maintain NMS, head posture, and postural balance in older adults.

## 1. Introduction

Aging is associated with impaired balance, which has been implicated in increasing the risk of falls [1]. Falls are associated with significant morbidity and mortality in older adults, and are the most common cause of accidental death and nonfatal injury in adults aged 65 years and older [2]. In older adults, many factors such as vision, proprioception, vestibular function, leg muscle strength, limitations in mobility, and use of medication affect their ability to maintain balance [3], which subsequently affects their quality of life [4].

Postural balance can be improved by performing the act of chewing [5]. In addition, chewing habit is correlated with forward head posture (FHP) [6], and a good oral health retains masticatory efficiency and postural muscular function (leading to good balance) in older adults [7,8,9]. Hence, tooth loss is a risk factor for postural imbalance [10], and dental occlusion is associated with static and dynamic balance [10,11]. Complete denture usage decreases masticatory function in older adults [12]. However, a previous study showed that denture use for a short period improves body balance compared to that of non-denture use [13,14]. Therefore, denture use may be related to balance in this population. Altered head posture and decreased craniovertical angle (CVA) was observed in adults who used dentures [15,16]. A smaller CVA was associated with a forward head position. A previous study reported that the FHP may worsen the static balance control [17,18,19]. A flexed posture is correlated with physical performance as well as quality of life [20,21].

There have been studies on the correlation between denture use and static balance; however, studies on the correlation between denture use in older adults and FHP, neck muscle strength, and postural balance are lacking.

Considering previous studies [13,14,17,18], the present study aimed to compare FHP, neck muscle strength, and balance between denture use group and non-denture use group in community-dwelling older adults. Furthermore, we also determined the correlation between duration of denture use and the factors above in the denture use group. We hypothesized that the denture use group had increased FHP, lower neck strength, and decreased balance compared to the non-denture use group, and that the duration of denture use was correlated with FHP, neck strength, and dynamic and static balance.

## 2. Materials and Methods

### 2.1. Ethical Considerations and Study Design

This study was conducted in accordance with the guidelines of the Declaration of Helsinki. All participants were informed in detail about the study procedure and safety, and they provided written informed consent. All study procedures were approved by Gachon University’s Institutional Review Board (1044396-201608-HR-062-01). This cross-sectional study used convenience sampling to recruit participants from the community-dwelling older adult population in Incheon, South Korea. The data were collected from September to December 2016.

### 2.2. Participants and Procedures

In this study, 107 older adults (age range 65–84 years) were recruited at a community center through advertisements (posters). Participants were selected via telephonic interviews according to the inclusion and exclusion criteria. The inclusion criteria were as follows: (1) wearing complete maxillary or mandibular dentures or wearing no dentures at all; (2) living independently and actively; and (3) having no history of balance disorder. While, exclusion criteria were, (1) experiencing pain during chewing; (2) having musculoskeletal or nervous system diseases that would interfere in the measurement of postural balance; and (3) having contraindications to any of the measurement procedures. The ages of the participants were matched between the two groups because denture use is partially dependent on the age.

The status of denture wear and functional teeth of the participants were examined by two specially trained dental hygienists who were blinded to the purpose of the study. The number of functional teeth more strongly predicts all-cause mortality than the number of residual teeth among community-dwelling older adults [22]. Hence, the functional teeth were evaluated in this study. Subsequently, we measured their forward head angle, neck muscle strength, and postural balance. For postural balance, static balance was measured after timed up-and-go test (TUG) measurement. The data were collected at senior community centers between September and October, and at a university laboratory between November and December. This study used G*Power software version 3.1.7 to calculate the sample size, which was determined based on a one-tailed t-test, power = 0.80, α = 0.05, and effect size = 0.5. In the comparison between the two groups, the effect size of 0.5 was used as the medium [23]. The required sample size was determined to be 102; thus, 107 participants were recruited.

### 2.3. Measurements

#### 2.3.1. Neck Muscle Strength

Neck muscle strength was assessed using a Stabilizer Pressure Biofeedback Unit (PBU, Chattanooga Group, Hixon TN, USA). The PBU comprises of a combined gauge/inflation bulb connected to a pressure cell. It is a simple device that registers pressure changes in an air-filled pressure cell, thereby allowing body movements, especially spinal movement. Participants were asked to lie down flat on their backs with their feet flat on the surface and knees bent, and the PBU was placed between the occiput and level of C7. Next, they were asked to pull their chins toward their chests while applying maximal pressure against the PBU. This method measured the strength of the deep cervical flexor muscle [24]. The inter-rater reliability of this method was 0.91 (95% confidence interval, 0.83–0.96) [25]. The measurement was repeated three times and the mean of these values was used for data analysis.

#### 2.3.2. FHP Assessment

FHP was measured using the CVA. For measuring FHP, all participants were made to sit on a backless chair and place their hands on their knees. While seated, they were instructed to look at points that were horizontal to the participant’s vision. To measure this angle, the fulcrum of a universal goniometer was placed on the C7 landmark, with the stationary arm positioned horizontal to the C7 landmark and the moving arm running parallel to the tragus [26]. Previous studies have indicated that CVA measurements have good test-retest reliability and high stability reliability, with reported intra-class correlation coefficients of 0.98 and 0.92–0.95, respectively [26]. The measurement was repeated three times, and the mean of these values was used for data analysis. For community-dwelling adults aged 65–85 years, angles of 39–46 degrees have been considered normal FHP [26]. Thus, for this study, abnormal FHP was defined as angles < 49 degrees [26].

#### 2.3.3. Balance

Dynamic and static balances were measured. After measuring the static balance, the dynamic balance was measured.

Dynamic balance for TUG. The TUG test is commonly used to assess functional mobility in community-dwelling older adults [27,28]. It was originally developed as a clinical measurement of dynamic balance in older people. It has a sensitivity of 92–93% for detection of clinically relevant balance problems [29]. Each participant was instructed to sit on a chair with no armrests. Upon receiving a voice signal, they were to stand up and walk 3 m along a line on the floor at a comfortable speed, then walk back to the chair and sit down. The time required to perform the TUG was measured, and the mean of three measurements was used.

Static balance for postural sway. Postural sway was assessed based on the measurement of center of pressure (COP) displacement during a static balance test. The participants were instructed to stand upright, barefoot, on a force sensor platform (Zebris FDM-S pressure platform, Zebris Medical GmbH, Germany) with their eyes open and gazing straight ahead. The force platform area measured 3.2 × 6.8 m and contained 2560 individually calibrated capacitive force sensors of approximately 0.85 × 0.85 cm, each positioned underneath the platform and sampled at 60 Hz. This allowed for an analysis of the distribution of static forces beneath the participant’s feet while standing on the platform. MR 3.8 software package (Noraxon Inc., Scottsdale, AZ, USA) was used to combine and process COP measurements and present them graphically and numerically on a monitor. The measured postural sway parameters were as follows: anteroposterior (AP) and mediolateral (ML) COP displacement, COP sway length, and velocity.

### 2.4. Statistical Analyses

All statistical analysis was conducted using SPSS version 24.0 (IBM Corp., Armonk, NY, USA). Analysis of frequency, descriptive statistics, and Chi-squared test were used to describe the participants’ general characteristics. The normality of the continuous variables was tested using Kolmogorov–Smirnov test. Intra-group comparisons were performed using independent sample *t*-test. In all participants, the correlation between denture use and dependent variables and whether duration of denture use was significantly correlated with the measured variables in the denture use group was evaluated using the Pearson correlation analysis, with the correlation between variables being expressed by Pearson’s r. Outcome variables are presented as mean ± standard deviation. Significance level was set at *p* < 0.05.

## 3. Results

Table 1 provides an overview of the participants’ general characteristics. The mean age of the 51 participants in the denture use group was 73.97 years, while that of the 56 participants in the non-denture use group was 72.45 years.

Compared with the non-denture use group, the denture use group had significantly lower neck muscle strength (*p* = 0.003) and a smaller craniocervical angle (*p* < 0.001), as well as a longer TUG (*p* < 0.001), COP sway length (*p* = 0.021), and AP COP displacement (*p* < 0.001) (Table 2).

**Figure 1:**
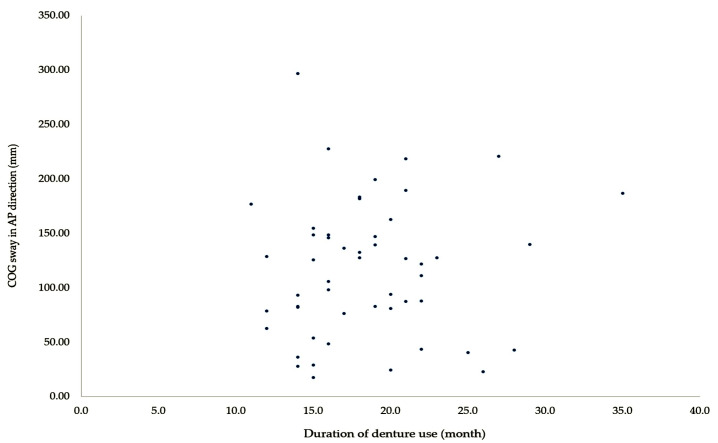
Scatter plot comparing duration of denture use and AP COP sway.

**Figure 2:**
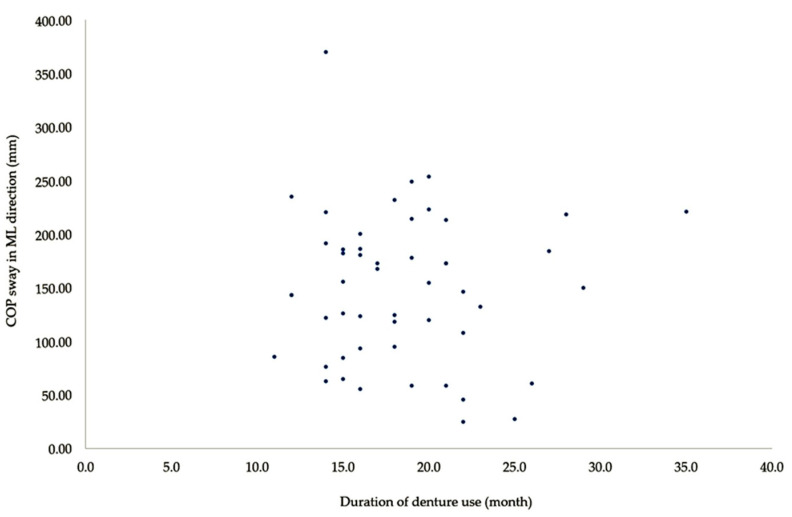
Scatter plot comparing duration of denture use and ML COP sway.

In the denture use group, duration of denture use was significantly correlated with TUG (*r* = 0.794, *p* < 0.001), AP COP sway (*r* = 0.278, *p* = 0.048), and ML COP sway (*r* = 0.277, *p* = 0.049) (Table 3). As shown in Figure 1 and Figure 2, there was no correlation between the two variables on a scatter plot. These results show that the duration of denture use is correlated with dynamic balance in older adults.

## 4. Discussion

In this study, the denture use group had decreased neck muscle strength, increased FHP, TUG, and postural sway than that of the non-denture use group. In addition, in the denture use group, the duration of denture use was correlated with the results of the TUG test.

In older adults, denture use is associated with reduced oral sensory function [30] and decreased CVA [31]. The CVA for the denture use group was 39.04 degrees, indicating excessive FHP. In addition, the denture use group had lower neck muscle strength than the non-denture group. These findings support the previously described association between denture use and natural head position in older adults [32].

The longer the duration of denture use, the lower the mastication efficiency and neck muscle activation [33]. In this study, the mean duration of denture use was 12.64 years. There is a positive functional association between the muscles of mastication and the activation of neck muscles [34]. This study also indicates that denture use may be associated with neck muscle weakness and FHP [35]. In addition, the average number of functional teeth was 26.05 for the non-denture use group and 9.67 for the denture use group. Zhang et al. [36] suggested that denture-users with ≤20 teeth also showed higher odds of being frail compared to those with >20 teeth. These results suggest that dentures are less efficient than natural teeth [36]. Therefore, the authors suggested that the use of dentures may reduce the masticatory efficiency, resulting in weakening of the flexor muscles of the neck, further leading to FHP. There is a disagreement in the previous reports on the correlation between denture use on musculoskeletal fragility [36,37]; hence, authors suggest that further research on this is needed in the future.

The coordination of the muscles of mastication, posture control (head and trunk), and those in the lower extremities has been reported during voluntary maximum clenching in dentate subjects [37]. While using complete dentures, the masticatory ability may be associated with static balance [12]. The results of our study indicated significant differences in COP sway length and AP COP displacement between the denture use and non-denture use groups. Another study identified a change in the activity of lower extremity muscles (rectus femoris and gastrocnemius) in denture wearers [38]. Specifically, because the gastrocnemius muscle requires minimal muscular effort to exert postural control during static upright standing [39], reduced activity in this muscle may indicate impaired postural control, explaining the increased postural sway [39]. Therefore, as we predicted in the hypothesis, postural sway increases with decrease in static balance in the denture use group due to changes in the activity of these muscles in this population. Further, the results of our study showed that the denture use group took 10.06 sec to complete the TUG test, whereas the non-denture-wearing group took only 8.66 sec. Accordingly, this test exhibited a significant correlation with denture use status. The TUG test is sensitive and specific for identifying community-dwelling older adults who are at risk for falls [29]. Our findings showed that the denture use can increase the TUG and AP COP displacement, and that the duration of denture use is related to TUG. In a previous study, denture use was shown to be effective in maintaining and improving the balance in older adults [13,14,36]. These studies showed that denture use confirmed the immediate change in postural sway and body balance, and report that denture use improved body balance. However, we obtained contradictory results. In this study, the TUG increased in the elderly who used denture for 12.64 years. Therefore, short-term denture use was found to have a positive effect on balance; conversely, long-term denture use may decrease balance. Since studies on the correlation between long-term denture use and balance are lacking, we propose the need for this research.

Our finding indicates that denture use does not help maintain and improve musculoskeletal fragility and postural balance. In addition, since the aging process leads to a deterioration of postural balance or musculoskeletal fragility, future studies are needed to explain the specific mechanisms underlying denture use and its association with musculoskeletal fragility and postural balance.

This study has some limitations. First, we recruited older adults wearing either maxillary or mandibular dentures, hindering the generalization of our findings to individuals wearing a complete set of dentures. Second, although denture wear correlates with mastication [40], in the current study, masticatory muscle activity was not considered. Third, for examining the main effect of denture use, precise status of the denture use must be analyzed. However, precise status of the denture use was not considered in this study. Fourth, this was a single center study. Finally, matching the number of residual teeth between the denture use and the non-denture use groups will be effective in identifying the relationship of use of denture and postural balance, NMS, and head posture. Future studies are needed to address these limitations.

Despite these limitations, this study has several advantages. This study is the first study to confirm the difference in neck muscle strength, head posture, and postural balance between those who use dentures and those who do not. Therefore, it provides the basis for further research on identifying the relationship of status of denture use and physical ability, and the number of remaining teeth.

## 5. Conclusions

In conclusion, we found that the denture use decreased neck muscle strength, increased FHP, and decreased static and dynamic balance. In addition, a significant relationship was found between duration of denture use and dynamic balance. Our finding suggested that long-term denture use may be an important factor in poor balance. Indeed, those results may increase awareness for health care professionals, including geriatric, since prolonged use of dentures might indicate a transition towards poor balance.

## Figures and Tables

**Table 1:** Baseline demographic characteristics of the participants.

	Denture Use (*n* = 51)	Non-Denture Use (*n* = 56)	*p*
Sex (male/female)	19/32	26/30	0.371 ^a^
Age (years)	73.78 ± 3.94	72.57 ± 4.59	0.147 ^b^
65–74 years old (*n*)	29 (56.9%)	32 (60.7%)	
74–84 years old (*n*)	22 (43.1%)	24 (39.3%)	
Height (cm)	157.80 ± 7.82	156.76 ± 7.22	0.474 ^b^
Weight (kg)	62.14 ± 11.84	61.03 ± 10.36	0.606 ^b^
BMI (%)	24.92 ± 4.18	24.76 ± 3.25	0.826 ^b^
Functional teeth (*n*)	9.67 ± 1.52	26.05 ± 1.175	<0.001 ^b^
Denture location maxilla mandibular	38 (74.5%) 13 (25.5%)		
Duration of denture use (years)	12.64 ± 4.39		

^a^ Statistical analysis was performed using Chi-squared test. ^b^ Statistical analysis was performed using independent t-test. BMI: body mass index. Values are expressed as mean ± standard deviation or n (%).

**Table 2:** Neck muscle strength, forward head pose (FHP), and balance comparison between denture use and non-denture use groups.

Variables (Unit)	Denture Use (*n* = 51)	Non-Denture Use (*n* = 56)	*p*	Difference (95% CI)
Neck muscle strength (mmHg)	37.88 ± 6.27	42.26± 8.46	0.003	4.388 (1.509–7.267)
Craniocervical angle (degree)	39.04 ± 7.48	49.43 ± 5.40	<0.001	10.391 (7.906–12.877)
TUG (sec)	10.06 ± 1.76	8.66 ± 1.42	<0.001	−1.398 (−2.008–0.787)
Postural sway parameters (unit: mm)				
COP sway length	379.90 ± 293.06	259.95 ± 234.99	0.021	−119.977 (51.146–221.362)
COP sway velocity	163.57 ± 120.38	164.47 ± 131.51	0.971	−0.901 (24.454–47.587)
COP sway in AP direction	115.92 ± 62.20	74.80 ± 41.83	<0.001	−41.116 (10.167–61.276)
COP sway in ML direction	148.58 ± 69.38	131.15 ± 61.50	0.171	−17.428 (12.653–42.518)

Statistical analysis was performed using independent *t*-test. TUG: timed up-and-go test, COP: center of pressure, AP: anteroposterior, ML: mediolateral.

**Table 3:** Correlation between duration of denture use and neck muscle strength, FHP, and postural balance in denture use group (*n* = 51).

Variables	Pearson Correlation Coefficient	*p*
Duration of denture use vs. TUG	0.794	<0.001
Duration of denture use vs. COP sway in AP direction	0.278	0.048
Duration of denture use vs. COP sway in ML direction	0.277	0.049

Pearson correlation Coefficient (*r*) and *p*-values are shown. TUG: timed up-and-go test, COP: center of pressure, AP: anteroposterior, ML: mediolateral.
